# Supplementary material for: Mechanisms governing the pioneering and redistribution capabilities of the non-classical pioneer PU.1
Source: Nat Commun. 2020 Jan 21;11:402. doi: 10.1038/s41467-019-13960-2 (PMC6972792; doi:10.1038/s41467-019-13960-2)
Supplement: Supplementary file 7 — Source data [file 41467_2019_13960_MOESM7_ESM.zip › Source_Data/FigureS2/FigureS2B_MotifScanOutput/KmeansCluster2/homerResults.html]

/misc/data/analysis/project\_PU1/CTV1/motifs/basic/PU1\_Kmeans14.2/final - Homer de novo Motif Results


# Homer *de novo* Motif Results (/misc/data/analysis/project\_PU1/CTV1/motifs/basic/PU1\_Kmeans14.2/final)

Known Motif Enrichment Results  
Gene Ontology Enrichment Results  
If Homer is having trouble matching a motif to a known motif, try copy/pasting the matrix file into
STAMP  
More information on motif finding results: HOMER
| Description of Results
| Tips
  
Total target sequences = 5023  
Total background sequences = 40634  
\* - possible false positive  

|  |  |  |  |  |  |  |  |  |
| --- | --- | --- | --- | --- | --- | --- | --- | --- |
| Rank | Motif | P-value | log P-pvalue | % of Targets | % of Background | STD(Bg STD) | Best Match/Details | Motif File |
| 1 | C T G A C G T A C G T A C G T A C T A G T C G A C T A G A C T G C G T A C G T A T A C G G C A T T A C G T G C A | 1e-2879 | -6.630e+03 | 76.41% | 5.19% | 22.9bp (75.0bp) | SpiB(ETS)/OCILY3-SPIB-ChIP-Seq(GSE56857)/Homer(0.963) More Information \| Similar Motifs Found | motif file (matrix) |
| 2 | T C G A T G A C A C T G C T G A A T C G T A C G C G T A C G T A C T A G | 1e-914 | -2.107e+03 | 52.16% | 11.34% | 51.1bp (83.2bp) | ELF3(ETS)/PDAC-ELF3-ChIP-Seq(GSE64557)/Homer(0.791) More Information \| Similar Motifs Found | motif file (matrix) |
| 3 | A C G T A G T C A G T C A G T C A G T C A G C T A C G T | 1e-442 | -1.018e+03 | 60.24% | 27.71% | 46.9bp (80.5bp) | RO3G\_00049(RRM)/Rhizopus\_oryzae-RNCMPT00205-PBM/HughesRNA(0.775) More Information \| Similar Motifs Found | motif file (matrix) |
| 4 | C T G A T A C G C G T A C T A G T C A G T G C A C G T A T A C G C G T A C A T G C T A G T G C A C G T A C T A G | 1e-426 | -9.824e+02 | 33.72% | 9.12% | 48.1bp (72.3bp) | SPIC/MA0687.1/Jaspar(0.702) More Information \| Similar Motifs Found | motif file (matrix) |
| 5 | T C A G T C A G T C G A C T A G A C T G T C G A C T G A T C A G T C G A T C G A T C A G | 1e-412 | -9.499e+02 | 30.00% | 7.34% | 49.5bp (100.9bp) | Unknown4/Arabidopsis-Promoters/Homer(0.782) More Information \| Similar Motifs Found | motif file (matrix) |
| 6 | G T A C G A C T G A T C C G A T G C A T T G A C G T A C G C A T G A T C C G A T A C G T A G T C G A C T A C G T | 1e-155 | -3.581e+02 | 15.73% | 4.80% | 56.8bp (72.1bp) | PU.1(ETS)/ThioMac-PU.1-ChIP-Seq(GSE21512)/Homer(0.658) More Information \| Similar Motifs Found | motif file (matrix) |
| 7 | A T C G G A T C G C T A T C A G G C A T A C G T T A G C A G T C G C A T A T C G G C A T G C A T | 1e-122 | -2.809e+02 | 4.84% | 0.43% | 38.4bp (65.3bp) | Ets1-distal(ETS)/CD4+-PolII-ChIP-Seq(Barski\_et\_al.)/Homer(0.795) More Information \| Similar Motifs Found | motif file (matrix) |
| 8 | A G C T G A C T C A G T A T G C A C G T A T C G A C G T A C G T A G T C A G T C A G C T A G T C C G A T G C A T | 1e-118 | -2.719e+02 | 7.19% | 1.29% | 42.6bp (66.9bp) | ZNF189(Zf)/HEK293-ZNF189.GFP-ChIP-Seq(GSE58341)/Homer(0.800) More Information \| Similar Motifs Found | motif file (matrix) |
| 9 | C G T A A T C G G C A T A G C T G A T C C G A T A T C G A G T C C G A T G C A T A G C T G A C T | 1e-105 | -2.441e+02 | 8.68% | 2.15% | 50.2bp (70.8bp) | SPI1/MA0080.4/Jaspar(0.711) More Information \| Similar Motifs Found | motif file (matrix) |
| 10 | A G T C G A C T A G C T A G T C A G T C C G A T A G T C G T A C G A C T A T G C A T G C G C A T A G T C | 1e-103 | -2.385e+02 | 13.64% | 4.97% | 50.0bp (88.4bp) | SF2(RRM)/Drosophila\_melanogaster-RNCMPT00066-PBM/HughesRNA(0.708) More Information \| Similar Motifs Found | motif file (matrix) |
| 11 | T C G A A G T C G A C T C G A T T A G C A G C T T A C G G A C T G C T A | 1e-103 | -2.375e+02 | 10.65% | 3.24% | 49.3bp (75.6bp) | PB0058.1\_Sfpi1\_1/Jaspar(0.695) More Information \| Similar Motifs Found | motif file (matrix) |
| 12 | C T G A C T G A C T G A T C A G C T G A A C T G C T A G C G T A C G T A T A C G A G T C G T A C G C T A | 1e-90 | -2.075e+02 | 4.60% | 0.64% | 38.5bp (77.3bp) | GCR1/Literature(Harbison)/Yeast(0.815) More Information \| Similar Motifs Found | motif file (matrix) |
| 13 | C G A T T A C G C T G A A C T G A T C G C G T A C T G A G T A C G C A T C T A G A T G C G T A C G T C A | 1e-76 | -1.759e+02 | 5.55% | 1.22% | 53.4bp (76.0bp) | SPIB/MA0081.1/Jaspar(0.737) More Information \| Similar Motifs Found | motif file (matrix) |
| 14 | T C A G A T G C T C G A C G T A C T A G C T A G A C T G C T A G A T C G C G T A G T C A | 1e-69 | -1.600e+02 | 7.21% | 2.18% | 54.2bp (80.4bp) | RO3G\_00049(RRM)/Rhizopus\_oryzae-RNCMPT00205-PBM/HughesRNA(0.700) More Information \| Similar Motifs Found | motif file (matrix) |
| 15 | A C T G C T G A A C T G C G T A C G T A A T G C A C G T | 1e-67 | -1.557e+02 | 22.58% | 12.95% | 50.2bp (76.5bp) | LIN28A(CSD,Znf)/Homo\_sapiens-RNCMPT00036-PBM/HughesRNA(0.772) More Information \| Similar Motifs Found | motif file (matrix) |
| 16 | A C G T A C G T A C G T A C T G C G T A A C T G A C T G C G T A C G T A A G T C | 1e-65 | -1.498e+02 | 1.83% | 0.06% | 50.3bp (50.8bp) | PB0162.1\_Sfpi1\_2/Jaspar(0.727) More Information \| Similar Motifs Found | motif file (matrix) |
| 17 | A G T C G C A T T A G C G A T C G C A T C A G T A T G C A G C T T A G C G A T C G C A T C A G T A T G C G C A T | 1e-64 | -1.484e+02 | 9.06% | 3.40% | 48.9bp (73.7bp) | RBM5(RRM)/Homo\_sapiens-RNCMPT00154-PBM/HughesRNA(0.672) More Information \| Similar Motifs Found | motif file (matrix) |
| 18 | A C T G A G T C C G T A A T G C C G T A C G T A G T C A | 1e-60 | -1.404e+02 | 44.93% | 32.98% | 57.8bp (69.5bp) | CPO(RRM)/Drosophila\_melanogaster-RNCMPT00133-PBM/HughesRNA(0.829) More Information \| Similar Motifs Found | motif file (matrix) |
| 19 | C A G T T A C G G A T C C G A T A T G C A G T C A G C T A G T C G T A C T G A C | 1e-59 | -1.367e+02 | 7.76% | 2.77% | 49.9bp (77.6bp) | NCU02404(RRM)/Neurospora\_crassa-RNCMPT00238-PBM/HughesRNA(0.854) More Information \| Similar Motifs Found | motif file (matrix) |
| 20 | C A T G C T G A T C G A C T G A A G T C C A G T A C G T C T G A G A T C T C G A C T G A A C G T T A G C | 1e-55 | -1.268e+02 | 2.29% | 0.22% | 73.2bp (68.3bp) | tll/dmmpmm(Papatsenko)/fly(0.622) More Information \| Similar Motifs Found | motif file (matrix) |
| 21 | T C G A G C A T A C T G C G T A C G T A A C T G C T A G C T G A C T G A A C T G C G T A T A C G | 1e-54 | -1.245e+02 | 4.66% | 1.20% | 53.7bp (79.6bp) | RBM5(Znf)/Homo\_sapiens-RNCMPT00055-PBM/HughesRNA(0.743) More Information \| Similar Motifs Found | motif file (matrix) |
| 22 | A C T G A C T G T G C A C G T A T C A G T C G A G T C A T C G A C T G A A C T G T A C G G T C A C G T A C T A G | 1e-53 | -1.229e+02 | 5.97% | 1.91% | 50.6bp (68.8bp) | GCR2/MA0305.1/Jaspar(0.621) More Information \| Similar Motifs Found | motif file (matrix) |
| 23 | C A T G C T G A C T A G A C T G T C G A C G T A C T A G A C T G T C G A C T G A C T A G T C A G | 1e-53 | -1.222e+02 | 6.73% | 2.35% | 52.4bp (89.8bp) | RBM5(Znf)/Homo\_sapiens-RNCMPT00055-PBM/HughesRNA(0.689) More Information \| Similar Motifs Found | motif file (matrix) |
| 24 | C G T A A C T G C G A T A C G T A G T C A C G T A G T C C T G A A G C T C T A G C G T A A C T G C G T A | 1e-50 | -1.159e+02 | 2.99% | 0.51% | 42.3bp (69.9bp) | ZBTB33(Zf)/GM12878-ZBTB33-ChIP-Seq(GSE32465)/Homer(0.701) More Information \| Similar Motifs Found | motif file (matrix) |
| 25 | A C G T A T C G A G T C C G A T C A T G G A T C G C A T C A T G A G T C | 1e-49 | -1.148e+02 | 29.42% | 19.99% | 54.9bp (73.0bp) | Adf1/dmmpmm(Bergman)/fly(0.669) More Information \| Similar Motifs Found | motif file (matrix) |
| 26 | A G T C T C G A G A C T C T A G T C G A C A G T A G T C A G T C T C G A T C G A C G A T A G T C C G T A A G T C | 1e-46 | -1.066e+02 | 1.39% | 0.06% | 79.0bp (55.8bp) | PH0014.1\_Cphx/Jaspar(0.768) More Information \| Similar Motifs Found | motif file (matrix) |
| 27 | G T A C G T A C G T A C G T C A A T G C C G T A A G T C C G A T A G C T A G T C A G T C C G A T G T A C G T A C | 1e-45 | -1.040e+02 | 1.47% | 0.08% | 38.5bp (53.1bp) | PU.1(ETS)/ThioMac-PU.1-ChIP-Seq(GSE21512)/Homer(0.778) More Information \| Similar Motifs Found | motif file (matrix) |
| 28 | C G T A G T A C C T G A C A T G T A C G G T C A C T G A C T A G G C T A C G T A C T A G G T C A C G T A C T G A | 1e-43 | -9.922e+01 | 4.36% | 1.29% | 50.7bp (73.2bp) | ETS:E-box(ETS,bHLH)/HPC7-Scl-ChIP-Seq(GSE22178)/Homer(0.699) More Information \| Similar Motifs Found | motif file (matrix) |
| 29 | A C G T G A T C A C G T A G T C T C A G A C G T C T A G C G T A G A C T C T G A C T A G G A C T C T A G | 1e-42 | -9.792e+01 | 2.11% | 0.28% | 52.3bp (64.7bp) | FZF1/MA0298.1/Jaspar(0.695) More Information \| Similar Motifs Found | motif file (matrix) |
| 30 | T G C A A G T C C G T A A G T C G A C T A G C T G C A T G A C T C G T A C T G A T C G A G A T C G T A C G C T A | 1e-42 | -9.754e+01 | 1.83% | 0.20% | 29.9bp (59.7bp) | AT1G69570(C2C2dof)/col-AT1G69570-DAP-Seq(GSE60143)/Homer(0.681) More Information \| Similar Motifs Found | motif file (matrix) |
| 31 | G A T C T G A C A G T C G C T A A C G T C T A G C G T A C G A T G A T C A G T C G C T A | 1e-34 | -7.841e+01 | 2.17% | 0.41% | 72.7bp (68.0bp) | ZNF143\|STAF(Zf)/CUTLL-ZNF143-ChIP-Seq(GSE29600)/Homer(0.713) More Information \| Similar Motifs Found | motif file (matrix) |
| 32 | G T C A A C T G A C G T A C G T A C G T A G T C A G T C A G C T A C T G C G T A | 1e-32 | -7.567e+01 | 1.81% | 0.29% | 78.2bp (67.5bp) | EWS:FLI1-fusion(ETS)/SK\_N\_MC-EWS:FLI1-ChIP-Seq(SRA014231)/Homer(0.758) More Information \| Similar Motifs Found | motif file (matrix) |
| 33 | A C G T A T G C A G C T C G T A A G T C G T A C T G A C A G T C A C G T A C G T A G T C A G T C G T A C | 1e-32 | -7.389e+01 | 1.49% | 0.18% | 37.3bp (62.2bp) | PCBP2(KH)/Homo\_sapiens-RNCMPT00044-PBM/HughesRNA(0.709) More Information \| Similar Motifs Found | motif file (matrix) |
| 34 | C G T A C T G A A T C G G T C A C T G A A T C G G T C A C G T A T A G C G T C A C G T A A C T G C G T A T C G A | 1e-30 | -6.932e+01 | 2.13% | 0.46% | 57.1bp (28.7bp) | PABPN1(RRM)/Homo\_sapiens-RNCMPT00157-PBM/HughesRNA(0.644) More Information \| Similar Motifs Found | motif file (matrix) |
| 35 | C T A G C G T A A C T G C T A G A C G T A C T G C G T A A G C T A G C T A C T G | 1e-28 | -6.657e+01 | 1.71% | 0.30% | 78.8bp (67.0bp) | Dux/MA0611.1/Jaspar(0.738) More Information \| Similar Motifs Found | motif file (matrix) |
| 36 | C G T A A G T C A C G T A G T C C T G A A G T C A C G T C T G A A G C T A G T C | 1e-28 | -6.615e+01 | 1.71% | 0.30% | 49.3bp (73.1bp) | STZ(C2H2)/colamp-STZ-DAP-Seq(GSE60143)/Homer(0.716) More Information \| Similar Motifs Found | motif file (matrix) |
| 37 | A G T C C T A G A C G T C T A G C G T A G A C T C G A T C T A G C G A T C G T A | 1e-28 | -6.606e+01 | 1.97% | 0.41% | 69.2bp (64.7bp) | Dux/MA0611.1/Jaspar(0.769) More Information \| Similar Motifs Found | motif file (matrix) |
| 38 | T A C G C G T A A C T G C G T A C G A T G T A C C G A T A T G C C T G A A G C T | 1e-27 | -6.418e+01 | 4.74% | 1.97% | 45.2bp (71.7bp) | GATA14(C2C2gata)/col-GATA14-DAP-Seq(GSE60143)/Homer(0.849) More Information \| Similar Motifs Found | motif file (matrix) |
| 39 | A G T C G A C T A C G T A G T C A C G T C G A T A G T C G T C A A G T C C T G A C A G T C A T G C T A G | 1e-27 | -6.304e+01 | 1.83% | 0.37% | 42.0bp (72.8bp) | INO4/INO4\_YPD/4-INO4,37-INO2(Harbison)/Yeast(0.750) More Information \| Similar Motifs Found | motif file (matrix) |
| 40 | A C G T G T A C A G T C A G C T A C T G C G T A T C A G C T A G A G T C A T G C A C G T | 1e-27 | -6.233e+01 | 1.87% | 0.39% | 80.3bp (71.6bp) | ZFX(Zf)/mES-Zfx-ChIP-Seq(GSE11431)/Homer(0.711) More Information \| Similar Motifs Found | motif file (matrix) |
| 41 | C T G A A C G T G A T C G T C A A G T C A G T C A C G T A G T C A G T C A G T C C G T A A G T C A G T C C G T A | 1e-26 | -6.199e+01 | 0.74% | 0.02% | 84.7bp (39.1bp) | AtMYB84(MYB)/Arabidopsis thaliana/AthaMap(0.649) More Information \| Similar Motifs Found | motif file (matrix) |
| 42 | A C G T C G T A C G T A A C T G A C G T A C G T A C G T A G T C A G T C | 1e-26 | -6.196e+01 | 1.83% | 0.38% | 75.9bp (63.7bp) | EXC-7(RRM)/Caenorhabditis\_elegans-RNCMPT00014-PBM/HughesRNA(0.760) More Information \| Similar Motifs Found | motif file (matrix) |
| 43 | C A T G C G A T C T A G C T A G T C G A G T C A G A T C C G A T A C T G T A G C C G A T C T A G | 1e-25 | -5.786e+01 | 1.29% | 0.19% | 58.6bp (71.8bp) | ZNF317(Zf)/HEK293-ZNF317.GFP-ChIP-Seq(GSE58341)/Homer(0.710) More Information \| Similar Motifs Found | motif file (matrix) |
| 44 | A T G C T G C A G C A T C T A G T C A G A G C T C T G A T C A G T C G A C G T A T C A G T C A G | 1e-24 | -5.586e+01 | 3.11% | 1.10% | 63.0bp (71.9bp) | Tv\_0259(RRM)/Trichomonas\_vaginalis-RNCMPT00259-PBM/HughesRNA(0.660) More Information \| Similar Motifs Found | motif file (matrix) |
| 45 | G T C A A C G T T G A C G C T A A G C T C T A G C T A G A T G C C T A G C T A G | 1e-23 | -5.423e+01 | 2.55% | 0.80% | 67.2bp (71.4bp) | Tv\_0259(RRM)/Trichomonas\_vaginalis-RNCMPT00259-PBM/HughesRNA(0.808) More Information \| Similar Motifs Found | motif file (matrix) |
| 46 | C G A T A T G C G T A C A C G T T G C A A T G C A C G T G T A C C G A T A C T G A G T C C G T A C G A T A G T C | 1e-22 | -5.217e+01 | 1.17% | 0.17% | 42.5bp (56.4bp) | PB0114.1\_Egr1\_2/Jaspar(0.620) More Information \| Similar Motifs Found | motif file (matrix) |
| 47 | A T G C A G C T C G T A T C A G A G T C A G T C C T G A C A G T | 1e-22 | -5.202e+01 | 19.01% | 13.65% | 55.8bp (69.6bp) | NFIC/MA0161.1/Jaspar(0.737) More Information \| Similar Motifs Found | motif file (matrix) |
| 48 | G T C A A G T C C G T A G A C T A T G C A G T C A G C T C G A T C T A G A G T C G A T C C G T A | 1e-20 | -4.664e+01 | 0.72% | 0.05% | 58.4bp (69.5bp) | PB0181.1\_Spdef\_2/Jaspar(0.685) More Information \| Similar Motifs Found | motif file (matrix) |
| 49 | A C T G A C G T C G T A G T A C A C T G A G C T A C G T A C G T | 1e-20 | -4.638e+01 | 2.47% | 0.85% | 70.2bp (66.5bp) | Nr2e3/MA0164.1/Jaspar(0.724) More Information \| Similar Motifs Found | motif file (matrix) |
| 50 | C T G A C T A G G C T A C T A G G A C T A C G T T C G A T G A C G T A C G T C A A G C T G T C A G A C T | 1e-20 | -4.616e+01 | 0.78% | 0.07% | 56.9bp (41.8bp) | OLIG3/MA0827.1/Jaspar(0.657) More Information \| Similar Motifs Found | motif file (matrix) |
| 51 | T G A C G C T A C G A T C G T A G T A C C G A T T C A G A G C T A G C T A C G T C G A T G T A C | 1e-19 | -4.563e+01 | 1.65% | 0.42% | 54.1bp (56.7bp) | Pp\_0229(RRM)/Physcomitrella\_patens-RNCMPT00229-PBM/HughesRNA(0.705) More Information \| Similar Motifs Found | motif file (matrix) |
| 52 | C T G A C T G A C T A G A G T C G A T C A T G C A G C T G T C A T C A G T G A C G T A C C G T A | 1e-19 | -4.507e+01 | 1.65% | 0.43% | 52.7bp (62.0bp) | TRB2/MA1073.1/Jaspar(0.621) More Information \| Similar Motifs Found | motif file (matrix) |
| 53 | C G T A C G T A A C T G A C G T A T C G A C T G A C G T A C G T | 1e-18 | -4.262e+01 | 2.27% | 0.78% | 57.3bp (69.5bp) | PH0004.1\_Nkx3-2/Jaspar(0.817) More Information \| Similar Motifs Found | motif file (matrix) |
| 54 | A G T C C A T G G A C T C A G T G A C T C T G A T G C A G T C A | 1e-17 | -4.020e+01 | 7.27% | 4.36% | 55.6bp (65.8bp) | ct/MA0218.1/Jaspar(0.845) More Information \| Similar Motifs Found | motif file (matrix) |
| 55 | G A T C A G T C A C G T A C T G A T G C A C G T C A G T A T G C C T G A A G T C C G T A | 1e-17 | -3.950e+01 | 1.06% | 0.20% | 45.7bp (69.0bp) | Tb\_0251(RRM)/Trypanosoma\_brucei-RNCMPT00251-PBM/HughesRNA(0.691) More Information \| Similar Motifs Found | motif file (matrix) |
| 56 | A C T G A G T C C G A T A C T G A T G C A T G C C G T A | 1e-15 | -3.675e+01 | 13.58% | 9.71% | 54.7bp (67.1bp) | NF1-halfsite(CTF)/LNCaP-NF1-ChIP-Seq(Unpublished)/Homer(0.818) More Information \| Similar Motifs Found | motif file (matrix) |
| 57 | A C G T G T A C A C G T A G T C C T G A A G T C A C T G C G T A | 1e-15 | -3.625e+01 | 3.54% | 1.70% | 48.3bp (70.1bp) | ZBTB33/MA0527.1/Jaspar(0.813) More Information \| Similar Motifs Found | motif file (matrix) |
| 58 | G C T A T C A G G A T C A C G T C T A G A C G T G T C A G C T A A G T C A G T C C T G A G T C A A G C T | 1e-15 | -3.535e+01 | 0.40% | 0.01% | 38.2bp (29.4bp) | bZIP52(bZIP)/colamp-bZIP52-DAP-Seq(GSE60143)/Homer(0.679) More Information \| Similar Motifs Found | motif file (matrix) |
| 59 | G T A C C G T A C T A G T A C G T G A C T C A G C G T A C T A G C T G A C A T G C G T A | 1e-14 | -3.424e+01 | 1.69% | 0.56% | 46.8bp (74.4bp) | BPC1(BBRBPC)/colamp-BPC1-DAP-Seq(GSE60143)/Homer(0.743) More Information \| Similar Motifs Found | motif file (matrix) |
| 60 | A C G T C A T G G C A T C A G T A G T C T G C A T C G A T G C A G A T C C G T A | 1e-14 | -3.356e+01 | 1.45% | 0.44% | 62.7bp (56.6bp) | At2g41835(C2H2)/col-At2g41835-DAP-Seq(GSE60143)/Homer(0.790) More Information \| Similar Motifs Found | motif file (matrix) |
| 61 | C A G T C T A G A T C G G A T C C T G A A T C G G A C T A G C T T C G A | 1e-14 | -3.338e+01 | 6.13% | 3.70% | 56.7bp (63.3bp) | Myb/MA0100.2/Jaspar(0.817) More Information \| Similar Motifs Found | motif file (matrix) |
| 62 | G A T C G A C T A G T C C G A T T A C G G A T C G A C T A G C T A G T C A T G C A G C T G T A C G T A C A G C T | 1e-14 | -3.275e+01 | 1.08% | 0.26% | 47.6bp (66.3bp) | GCR2(MacIsaac)/Yeast(0.714) More Information \| Similar Motifs Found | motif file (matrix) |
| 63 | A G C T A C T G C T G A A C G T C G T A A C T G A C G T | 1e-14 | -3.253e+01 | 11.89% | 8.48% | 59.3bp (64.6bp) | FZF1/MA0298.1/Jaspar(0.880) More Information \| Similar Motifs Found | motif file (matrix) |
| 64 | A C G T C T G A G T A C A G T C C T G A A G C T C T G A C G A T A T C G C G T A | 1e-13 | -3.143e+01 | 1.02% | 0.24% | 52.5bp (63.7bp) | OLIG3/MA0827.1/Jaspar(0.767) More Information \| Similar Motifs Found | motif file (matrix) |
| 65 | A C G T C A T G C A G T G A T C A G C T A T C G G C T A G T C A A G T C T A C G | 1e-13 | -3.034e+01 | 0.98% | 0.23% | 50.9bp (71.9bp) | sma-4/MA0925.1/Jaspar(0.686) More Information \| Similar Motifs Found | motif file (matrix) |
| 66 | G A T C A G C T G T A C A G T C A C G T A G T C C G T A A C G T C T A G G A C T A G T C A C G T A T G C A G C T | 1e-12 | -2.989e+01 | 0.42% | 0.03% | 57.5bp (52.2bp) | PUF68(RRM)/Drosophila\_melanogaster-RNCMPT00141-PBM/HughesRNA(0.643) More Information \| Similar Motifs Found | motif file (matrix) |
| 67 | C G T A G A C T T A G C T G C A T A G C G T C A T C A G G A C T T C G A T C A G G T C A | 1e-12 | -2.948e+01 | 0.96% | 0.23% | 66.8bp (70.0bp) | Tb\_0252(RRM)/Trypanosoma\_brucei-RNCMPT00252-PBM/HughesRNA(0.648) More Information \| Similar Motifs Found | motif file (matrix) |
| 68 | A C G T T A G C A G C T A G T C C G A T A C G T A C T G C G T A A C T G C G A T A C G T A G T C | 1e-12 | -2.939e+01 | 0.82% | 0.16% | 54.8bp (76.5bp) | tin/dmmpmm(SeSiMCMC)/fly(0.695) More Information \| Similar Motifs Found | motif file (matrix) |
| 69 | A T C G G A T C A T G C A C G T C G A T C G T A G T A C A C G T A C G T A C T G C G T A | 1e-12 | -2.906e+01 | 0.40% | 0.02% | 55.0bp (34.8bp) | vnd/dmmpmm(Noyes\_hd)/fly(0.729) More Information \| Similar Motifs Found | motif file (matrix) |
